# Some of the Diseases of the Rectum and Their Homœopathic and Surgical Treatment

**Published:** 1884-10

**Authors:** 


**Some of the Diseases of the Rectum and their Homœopathic and Surgical Treatment.** By MORTIMER AYRES, M. D. Chicago: Duncan Bros. 1884.

A little book of 75 pages was written, as the preface says, because the author had become possessed of some facts that enabled him to manage them with satisfaction, or, in his own words, he had quite a repute for "curing piles." There is a good deal of practical information in the book.

